# Regulation of the Expression, Oligomerisation and Signaling of the Inhibitory Receptor CLEC12A by Cysteine Residues in the Stalk Region

**DOI:** 10.3390/ijms221910207

**Published:** 2021-09-22

**Authors:** Julien Vitry, Guillaume Paré, Andréa Murru, Xavier Charest-Morin, Halim Maaroufi, Kenneth R. McLeish, Paul H. Naccache, Maria J. Fernandes

**Affiliations:** 1 CHU de Québec Research Center, Division of Infectious Diseases and Immunology, Laval University, Québec, QC G1V 4G2, Canada; julien.vitry@crchudequebec.ulaval.ca (J.V.); guillaume.pare@crchudequebec.ulaval.ca (G.P.); andrea.murru@crchudequebec.ulaval.ca (A.M.); paul.naccache@crchudequebec.ulaval.ca (P.H.N.); 2 Department of Microbiology-Infectious Diseases and Immunology, Faculty of Medicine, Laval University, Québec, QC G1V 4G2, Canada; xav_c_moi@hotmail.com; 3 Institute of Integrative Biology and Systems, Laval University, Québec, QC G1V 0A6, Canada; Halim.Maaroufi@ibis.ulaval.ca; 4 Department of Medicine, University of Louisville School of Medicine, Louisville, KY 40206, USA; kenneth.mcleish@louisville.edu

**Keywords:** inhibitory receptor, C-type lectin receptor, CLEC12A, cysteine residues, stalk domain, oligomerisation, signaling, flotillin

## Abstract

CLEC12A is a myeloid inhibitory receptor that negatively regulates inflammation in mouse models of autoimmune and autoinflammatory arthritis. Reduced CLEC12A expression enhances myeloid cell activation and inflammation in CLEC12A knock-out mice with collagen antibody-induced or gout-like arthritis. Similarly to other C-type lectin receptors, CLEC12A harbours a stalk domain between its ligand binding and transmembrane domains. While it is presumed that the cysteines in the stalk domain have multimerisation properties, their role in CLEC12A expression and/or signaling remain unknown. We thus used site-directed mutagenesis to determine whether the stalk domain cysteines play a role in CLEC12A expression, internalisation, oligomerisation, and/or signaling. Mutation of C118 blocks CLEC12A transport through the secretory pathway diminishing its cell-surface expression. In contrast, mutating C130 does not affect CLEC12A cell-surface expression but increases its oligomerisation, inducing ligand-independent phosphorylation of the receptor. Moreover, we provide evidence that CLEC12A dimerisation is regulated in a redox-dependent manner. We also show that antibody-induced CLEC12A cross-linking induces flotillin oligomerisation in insoluble membrane domains in which CLEC12A signals. Taken together, these data indicate that the stalk cysteines in CLEC12A differentially modulate this inhibitory receptor’s expression, oligomerisation and signaling, suggestive of the regulation of CLEC12A in a redox-dependent manner during inflammation.

## 1. Introduction

The myeloid C-type lectin-like inhibitory receptor 12A (CLEC12A) regulates immune responses in various pathological contexts, including gout, rheumatoid arthritis, and viral infection [1,2,3,4,5]. In knock-out mouse models of gout and rheumatoid arthritis, a diminution in CLEC12A expression enhances inflammation and disease severity [6,7]. Similarly, low levels of CLEC12A expression by circulating neutrophils and monocytes from early rheumatoid arthritis patients correlate with higher disease activity [8]. On the other hand, interferon production is significantly downregulated in lymphocytic choriomeningitis virus infected CLEC12A KO mice, resulting in an increased viral load and liver damage [9]. Thus, CLEC12A differentially regulates myeloid cell responses in a stimulus-dependent manner.

CLEC12A has an extracellular C-type lectin-like domain (CTLD), the defining feature of C-type lectin receptors (CLRs) [10,11,12,13]. While CTLDs typically bind a diverse array of carbohydrate ligands and some have evolved to bind proteins, endogenous ligands that specifically bind the CLEC12A CTLD remain unidentified [10,11,12,13,14]. CLEC12A CTLD is linked to the transmembrane domain by a stalk region that mediates receptor oligomerisation in other CLRs [5] The short cytoplasmic tail of CLEC12A comprises an immunoreceptor tyrosine-based inhibitory motif (ITIM) through which it regulates intracellular signaling pathways.

The paradigm of inhibitory receptor signaling involves ligand-induced receptor clustering leading to ITIM phosphorylation and the recruitment of phosphatases that inhibit activation signal transduction pathways [15,16]. In the absence of a known natural ligand, antibody-induced CLEC12A clustering has identified key events in CLEC12A signaling. Antibody-mediated crosslinking induces CLEC12A translocation to flotillin-rich membrane domains, where ITIM is phosphorylated in a Src-dependent manner [17]. Phosphatases recruited by CLEC12A include SHP-1 and SHP-2 [5]. Signaling proteins with reduced phosphorylation after CLEC12A clustering and internalisation include components of the MAP kinase and PI3K-Akt pathways [17]. CLEC12A regulates the monosodium urate-crystal (MSU)-induced release of IL-8 by neutrophils through the p38/PI3K-Akt signaling pathway [17,18]. In addition to regulating cytokine release, reactive oxygen species production is enhanced in CLEC12A KO mice [9].

While the role of CLEC12A in several inflammatory diseases is well established and the signaling pathways it modulates are partially defined, little is known about the molecular determinants of CLEC12A function. A potentially essential domain for CLEC12A function is its stalk region as it harbours two cysteine residues. While it is presumed that these cysteine residues have receptor multimerisation properties, this has not been demonstrated experimentally. Herein, we tested the hypothesis that the two cysteine residues in the stalk domain of CLEC12A regulate its expression, internalisation, signaling and/or function through their ability to induce receptor oligomerisation.

## 2. Results

### 2.1. Construction of Mutant CLEC12A Receptors

**Figure 1:**
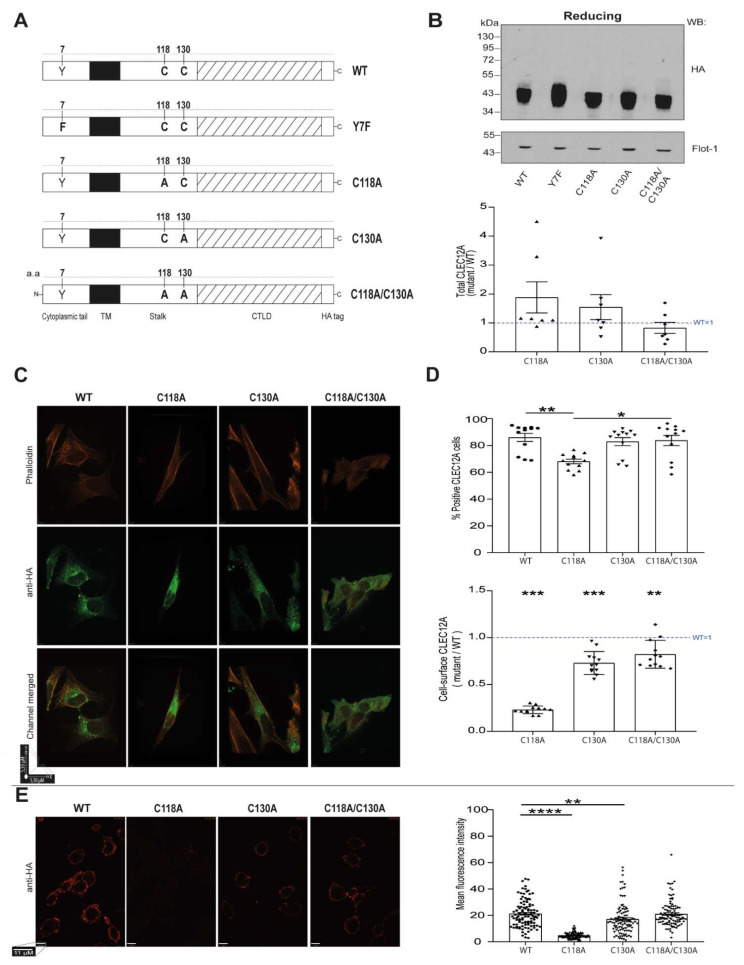
Stalk domain C118 and C130 residues regulate CLEC12A cell-surface expression and oligomerisation. (**A**) A schematic diagram of the constructs used in these experiments. Wild-type CLEC12A (WT), CLEC12A ITIM mutant (Y7F), and the C118A, C130A and C118A/C130A double mutants. All constructs are HA-tagged at the C-terminus. (**B**) Western blot of HeLa cells transiently transfected with the constructs was performed with an anti-HA and anti-flotillin-1 antibody *(loading control)*. Densitometry analysis of the Western blot is shown in the graph *below the gel*. (**C**) Confocal microscopy of HeLa cells transiently expressing the indicated constructs. Permeabilised cells were stained with an anti-HA antibody *(green)* and phalloidin *(red).* X and Y axis scale bar = 5, 30 µM. (**D**) Cell-surface, CLEC12A expression in HEK-293T cells transiently transfected with the constructs as determined by flow cytometry. The proportion of cells expressing the transfected CLEC12A plasmids is shown in the *upper graph* and the cell-surface expression is represented as a ratio over the level of expression of WT CLEC12A in the *bottom graph*. (**E**) Confocal microscopy of non-permeabilised HEK-293T cells transiently expressing the indicated constructs and stained with an anti-HA antibody *(red)*. Quantitation of CLEC12A cell-surface expression is shown in the *right graph* and expressed as the mean fluorescence intensity (MFI). Scale bar = 11 µM. The data in the Western blot in (**B**) is representative of seven independent experiments (*n* = 7) and flow cytometry in (**D**), of 12 independent experiments (*n* = 12). Confocal microscopy experiments in (**C**, **E**) are representative of three independent experiments (*n* = 3). The data in the graph in (**E**) was generated by analysing the MFI of 35 cells per experiment. Statistical analysis: *(upper* **D** *panel* and **E***)* Kruskal-Wallis test and multiple comparison Dunn’s test * *p* < 0.05; ** *p* < 0.01; *** *p* < 0.001; **** *p* < 0.0001. (**D** *bottom panel*) One sample Wilcoxon test ** *p* < 0.01; *** *p* < 0.001.

Cysteine residues are key for protein stability and function as they can form disulfide bonds within and between polypeptide chains [19]. CLEC12A contains two cysteine residues in its stalk region, C118 and C130, that are postulated to form disulfide bonds and lead to CLEC12A oligomerisation [5]. Sequence alignment revealed that these cysteine residues are conserved with CLEC-1 and CLEC12B [5]. suggestive of an important role for these residues in CLR function. We thus used a site-directed mutagenesis approach to probe the functional properties of the CLEC12A stalk domain cysteines. The mutant CLEC12A constructs generated are shown in Figure 1A. We substituted the cysteines with alanines and added a HA tag at the C-terminus to track their expression and to induce CLEC12A clustering. We previously reported that the addition of this tag does not interfere with CLEC12A expression and that CLEC12A-HA can be cross-linked with an anti-HA antibody to induced signaling in HEK-293T cells [17]. We also used a CLEC12A construct with a mutation in the ITIM tyrosine residue as a control.

### 2.2. Stalk Domain C118 and C130 Regulate CLEC12A Cell-Surface Expression and Oligomerisation

Since CLRs can oligomerise by forming disulfide bonds [14]. we determined the role of the stalk cysteine residues in CLEC12A expression and oligomerisation. The transient transfection of the CLEC12A constructs in HEK-293T cells revealed that CLEC12A WT, C118A, C130A and the double mutant C118A/C1130A are expressed at comparable levels by Western blot analysis (Figure 1B). Similarly, the transfection efficiency was comparable for all constructs (Figure 1B, *graph*). CLEC12A typically migrates in SDS-PAGE gels as a thick band of a molecular weight between 43–55 kDa, as it is highly glycosylated [18]. The mutant CLEC12A constructs migrated faster, suggesting that glycosylation is altered (Figure 1B). Confocal microscopy showed that the C118 mutant was predominantly expressed intracellularly with reduced plasma membrane expression (Figure 1C). This observation was confirmed by flow cytometry and confocal microscopy of non-permeabilised cells (Figure 1D,E, respectively). In contrast to the intracellular vesicular and cell-surface staining pattern of CLEC12A WT, C130A and the double mutant, the C118A mutant exhibited diffused cytoplasmic staining. We interpret those findings to indicate that C118 is essential for CLEC12A intracellular distribution and transport to the plasma membrane. With regards to receptor internalisation, the cysteine mutants did not alter the antibody-induced internalisation of CLEC12A (Supplementary Figure S1).

**Figure 2:**
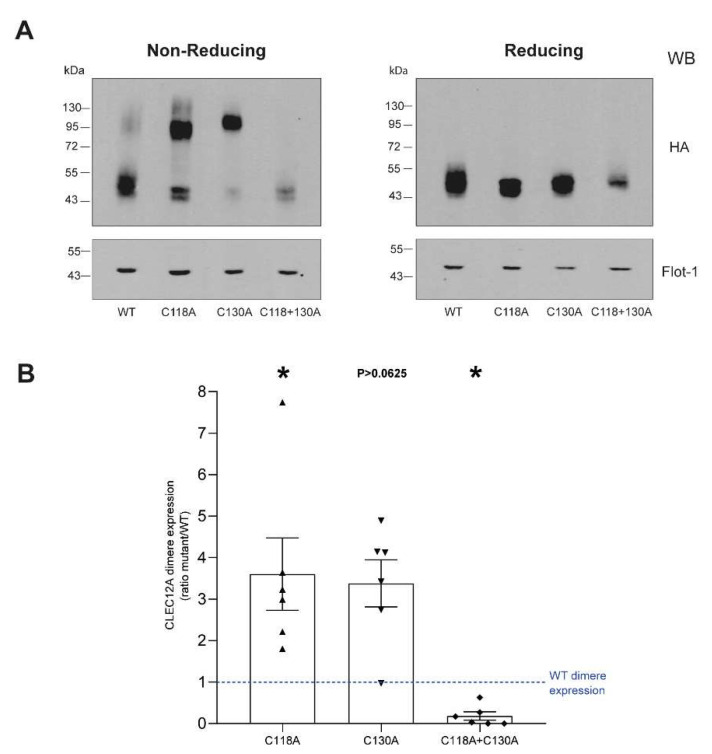
CLEC12A oligomerisation is dependent on C118 and C130. (**A**) Oligomerisation of WT CLEC12A and CLEC12A cysteine mutants was analyzed by Western blot of transiently transfected HEK-293T cells with the anti-HA and flotillin-1 antibody *(loading control)* under non-reducing *(left blot)* and reducing *(right blot)* conditions. (**B**) Densitometry analysis of CLEC12A oligomer expression in the non-reducing gel is shown as the following ratios: (CLEC12A mutant dimer/CLEC12A mutant dimer + monomer)/(CLEC12A WT dimer/CLEC12A WT dimer + monomer)’. The dotted line represents the CLEC12A WT ratio set to 1 for comparison with the CLEC12A mutants. Data in (**A**) are representative of six and three independent experiments for the non-reducing and reducing immunoblots, respectively. Statistical analysis: One sample Wilcoxon test * *p* < 0.05.

To determine the role of C118 and C130 in CLEC12A oligomerisation, we began by comparing the migration of CLEC12A WT to the double cysteine mutant under reducing and non-reducing conditions. Figure 2A shows that under non-reducing conditions, CLEC12A WT migrates as two bands. The higher molecular weight band is approximately 90–100 kDa, approximately twice the molecular weight of the lower band that corresponds to differentially glycosylated CLEC12A monomers. Under reducing conditions, CLEC12A WT migrates as a single band within the 43–55 kDa range, indicating that the upper 90 kDa band in the non-reducing gel represents a CLEC12A oligomer formed by disulfide bonding. A similar analysis of the double cysteine mutant revealed that disulfide bonding-dependent, CLEC12A oligomerisation occurs through C118 and/or C130. A comparison of the migration of C118A or C130A supported this observation. CLEC12A oligomerisation was observed when at least one of the two cysteine residues was present. Moreover, CLEC12A oligomerisation was significantly enhanced for both single cysteine mutants. Together, these data identify distinct roles for cysteine stalk residues in the regulation of CLEC12A cell-surface expression and oligomerisation.

### 2.3. CLEC12A Phosphorylation and Phosphatase Recruitment Is Regulated by C130

**Figure 3:**
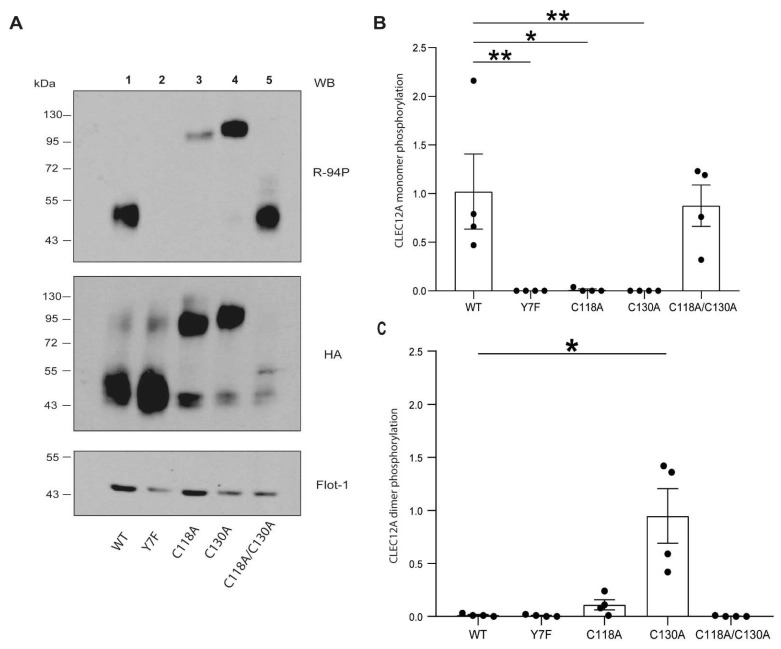
C118 and C130 mutants are phosphorylated. (**A**) CLEC12A phosphorylation was analyzed by Western blot with the R-94P antibody, the anti-HA antibody and the anti-Flotillin-1 *(loading control)* in HEK-293T cells transiently expressing the indicated CLEC12A constructs and treated with pervanadate. (**B**,**C**) Densitometry analysis of the phosphorylated monomer and dimer forms of WT and mutant CLEC12A. These data are representative of 4 independent experiments. Statistical analysis: Kruskal-Wallis, uncorrected Dunn’s test * *p* < 0.05; ** *p* < 0.01.

Inhibitory receptor signaling is initiated by receptor clustering resulting in ITIM phosphorylation [20]. The role of cysteine residues in the stalk region of CLEC12A on ITIM phosphorylation was examined in pervanadate-treated cells. Immunoblot analysis with an anti-phospho CLEC12A antibody showed that inhibition of protein tyrosine phosphatases with pervanadate resulted in CLEC12A WT phosphorylation in HEK-293T transfected cells (Figure 3A, *lane 1*). CLEC12A ITIM phosphorylation was detected with an in-house generated, anti-phospho CLEC12A ITIM antibody (R-94P) described in Paré et al. [20]. Phosphorylation was ITIM-specific as mutating this motif’s tyrosine to a phenylalanine (Y7F) abolished CLEC12A phosphorylation (Figure 3A, *lane 2*). The double cysteine CLEC12A mutant was also phosphorylated (Figure 3A, *lane 5*) indicative that C118 and C130 are not required for CLEC12A monomer phosphorylation in pervanadate-treated cells. In line with this observation, mutation of C118 or C130 did not abolish CLEC12A ITIM phosphorylation as R-94P bound both these CLEC12A mutants (Figure 3A, *lane 3 and 4*). Phosphorylation was, however, predominantly observed in C118A and C130A dimers. Moreover, there was a strong tendency for the level of phosphorylation to be superior for the C130A dimers than the C118A dimers but this difference did not reach significance.

**Figure 4:**
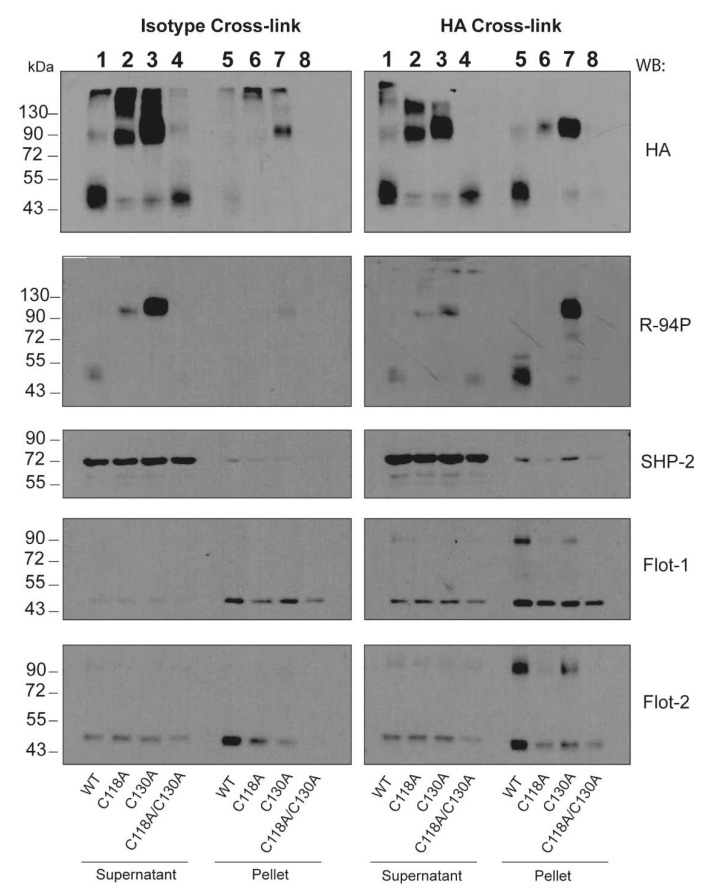
Involvement of neck domain C118 and C130 in early signaling initiated by antibody-induced clustering of CLEC12A. Wild-type (WT) and mutant CLEC12A expressed transiently in HEK-293T cells were cross-linked with an anti-HA or a mouse isotype (IgG) control antibody and a secondary (F(ab’)_2_ antibody prior to cell lysis with NP-40 buffer as described in *Materials and Methods.* CLEC12A translocation was analysed by Western blot of the supernatant (SN) or pellet (P) with the R-94P, anti-HA, anti-SHP-2 or anti-flotillin-1 and 2 antibodies. The flotillin-1 protein was used a loading control. These data are representative of three independent experiments.

To determine if the CLEC12A mutants are phosphorylated upon the antibody-induced clustering, the HA-tagged CLEC12A WT and mutants were cross-linked with an anti-HA and secondary antibody prior to Western blotting. As we previously reported [17], cross-linking induces the translocation of the CLEC12A WT monomer to the NP40 insoluble fraction (pellet) of the cell lysate (Figure 4, *compare lane 1 and 5 of the right, HA gel)*. This translocation is not observed in cells incubated with an isotype control antibody (Figure 4, *compare lane 1 and 5 of the left, HA gel)*. Blotting with R-94P confirmed our previous observation that the CLEC12A WT monomer is phosphorylated in the insoluble fraction of the lysate after cross-linking (Figure 4, *compare compare lane 1 and 5 of the right, R-94P gel*). In contrast, in the absence of both stalk cysteines, CLEC12A translocation is abolished and phosphorylation significantly reduced (Figure 4, *lane 4 and 8 of the right, HA and R-94P gels)*. Both C118A and C130A are sufficient, however, to preserve CLEC12A translocation in its dimer form (Figure 4, *compare lanes 2 and 6, and lanes 3 and 7 of the right, HA gel)*. Translocation of the C118A dimer is significantly inferior to that of C130A dimer, highly likely due to its low level of cell-surface expression. Strikingly, the C118A and C130A dimers in the supernatant fraction of cells incubated with the isotype antibody were constitutively phosphorylated. Increased receptor oligomerisation thus induces CLEC12A phosphorylation (Figure 4, *compare lane 2 and 3 to the WT in lane 1in the left, R-94P gel*). While cross-linking induced the translocation of C130A dimers to the pellet fraction, an increase in their phosphorylation was not apparent as their basal level of phosphorylation was already very high (Figure 4, *compare lanes 3 and 7 of the right, R-94P gel)*. Similar observations were made for C118A. Phosphorylation of the C118A mutant in the pellet was, however, significantly lower than C130A highly likely due to a significantly inferior amount of the C118A dimer in the pellet fraction compared to C130A.

We and others previously reported the recruitment of SHP-2 by CLEC12A after phosphorylation induced by antibody-mediated clustering or treatment with pervanadate [5,17]. In contrast, SHP-2 was not detected in the pellet fraction in cells expressing the double cysteine mutant (Figure 4, *lane 8 of the right, SHP-2 blot*). A similar observation was made for C118A, highly likely due to the low levels of cell-surface expression of this mutant receptor. As for C130A, it recruits SHP-2 to the pellet fraction after antibody-induced cross-linking similar to CLEC12A WT (Figure 4, *compare lanes 5 and 7 of the right, SHP-2 blot*). Together, these results indicate that the presence of the C118 residue is essential for SHP-2 recruitment by CLEC12A.

**Figure 5:**
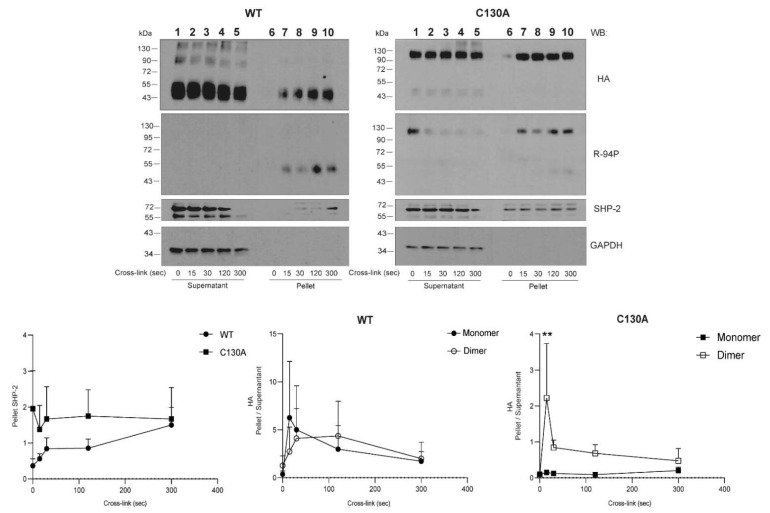
The C130 mutant enhances the recruitment of SHP-2 to the insoluble membrane fraction. Wild-type (WT) CLEC12A and C130 expressed transiently in HEK-293T cells were cross-linked with an anti-HA or a mouse isotype control antibody before cell lysis at the indicated time points as described in *Materials and Methods.* CLEC12A translocation from the NP40 soluble (supernatant (SN)) to insoluble pellet (P) was analysed by Western blot under non-reducing conditions with the anti-HA, R-94P, anti-SHP-2 and anti-GAPDH *(loading control)* antibodies. These data are representative of 3 independent experiments. Densitometry analysis was performed for both constructs as follows, at the indicated time points. The proportion of SHP-2 in the pellet relative to the amount of pellet CLEC12A-HA was calculated as the ratio of pellet (SHP-2/GADPH)/(anti-HA/GADPH) in the *left graph*. The proportion of oligomers and monomers that translocated to the pellet was calculated as the ratio of pellet anti-HA/supernatant anti-HA signal, shown in the *middle graph* for WT CLEC12A and C130A in the *right graph*. Statistical analysis: Uncorrected Fisher’s LSD test ** *p* < 0.01.

Signaling reactions are dynamic over time. As C130A was able to translocate to the insoluble fraction of the cell lysate to the same extent as CLEC12A WT, we examined the possibility that mutating C130 influences the kinetics of CLE12A translocation, phosphorylation, and/or SHP-2 recruitment. A time course experiment was performed to follow these molecular events by Western blot analysis at the indicated time points. We not only confirmed that C130A dimers translocated to the pellet after CLEC12A cross-linking, but also that this phenomenon occurred with faster kinetics than for CLEC12A WT monomers (Figure 5, *compare lanes 1–5 to 6–10 in C130A, HA gel*). The proportion of C130A dimers in the pellet at 15 s post-cross-linking was two-fold higher than CLEC12A WT monomers and already at its peak of recruitment to the pellet compared to 120 s for CLEC12A WT. Moreover, SHP-2 recruitment to the insoluble NP40 pellet is also accelerated in cells expressing C130A dimers as it is at its highest level a 15 s after cross-linking compared to 300 s for CLEC12A WT monomers (Figure 5, *compare lanes 6–10 in left and right anti-SHP-2 blots*). Together, these data indicate that C130A is a gain of function mutation.

### 2.4. Modeling of CLEC12A Mutants

**Figure 6:**
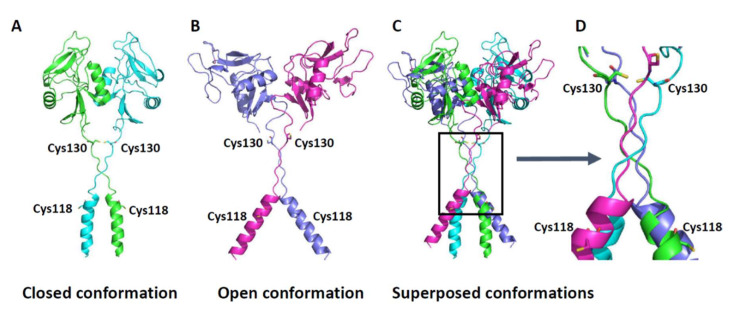
3D models of CLEC12A in different conformations. (**A**) CLEC12A in closed conformation with Cys118 and Cys130 that could form disulfide bridge between two monomer. (**B**) CLEC12A in open conformation with Cys118 and Cys130 that are predicted to not form disulfide bridge. (**C**) Superposition of closed and open conformation. (**D**) Close-up in the region containing Cys118 and Cys130. 3D models images were generated using PyMOL (http://www.pymol.org) (accessed on 1 February 2016).

To gain insight into the effect of C118A and C130A on the quaternary structure of CLEC12A, we generated a 3D model of CLEC12A with structural data of related CLRs Ly49 and ORL1. Figure 6A shows the predicted closed conformation of CLEC12A when both C118 and C130 form disulfide bonds and CLEC12A dimerizes. Our modelling predicts that the C118A mutant exhibits an open conformation as it does not form a disulfide bond (Figure 6B). In this open conformation, the cytoplasmic tails of CLEC12A are farther apart than in the closed conformation. Since receptor oligomerisation is a dynamic process, these CLEC12A conformations may co-exist. Non-reducing and reducing PAGE of C118, C130 and C118 + C130 mutations and 3D models suggest that there are three types of homodimers in equilibrium with monomers: C118-C118 disulfide bridge homodimer, C130-C130 homodimer, and C118-C118/C130-C130 homodimer. In addition, mutation of C118 promotes the formation of C130-C130 disulfide bridge homodimer and vice versa. Together, our experimental data indicate that disulfide bonding between C118 residues of adjacent CLEC12A polypeptides pushes the oligomerisation equilibrium towards the closed conformation, bringing the two CLEC12A polypeptides closer to each other to favor signaling via the cytoplasmic ITIM motif.

### 2.5. Regulation of CLEC12A Oligomerisation by Oxidation

**Figure 7:**
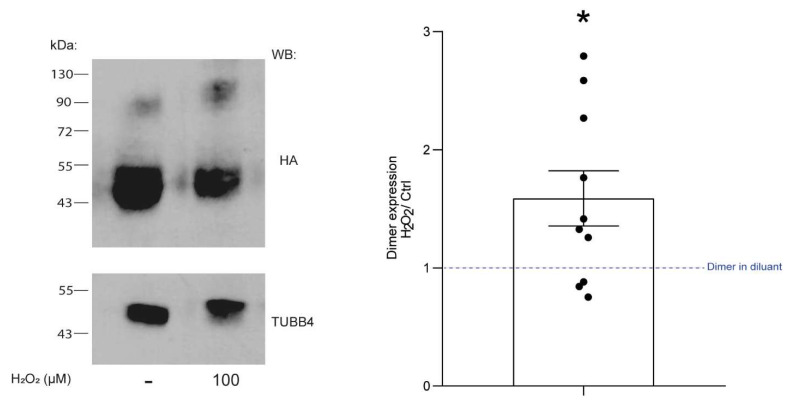
Oxidation enhances CLEC12A WT oligomerisation. HEK-293T cells transiently expressing CLEC12A WT were either incubated with 100 µM H_2_O_2_ or diluent (H_2_O) prior to Western blot analysis with an anti-HA and TUBB4 antibody *(loading control)*. Densitometric analysis of the Western blot data was performed by calculating the ratio of CLEC12A dimer signal after H_2_O_2_ incubation/CLEC12A dimer signal formed during incubation with the diluent. These data are representative of 10 independent experiments. Statistical analysis: One sample Wilcoxon test * *p* < 0.05.

Disulfide bonds form through an oxidation reaction involving cysteine residues [19,21]. We tested the hypothesis that oxidation would enhance the formation of CLEC12A oligomers by incubating cells transiently transfected with CLEC12A WT with H_2_O_2_ at a physiological level of 100 µM [22]. The ratio of CLEC12A WT oligomers to monomers formed in peroxide-treated HEK-293T cells transiently transfected with CLEC12A WT was significantly higher compared to cells incubated in buffer (Figure 7). These data suggest that CLEC12A stalk cysteines regulate CLEC12A oligomerisation, in part, by sensing the oxidative state of the environment.

### 2.6. Flotillin Regulates CLEC12A Phosphorylation

Flotillins are best known for their scaffolding properties that are crucial for the formation of signaling membrane platforms [23]. The proximity of receptors and signaling molecules in these protein complexes facilitates the initiation of cellular signaling to either promote or downregulate cellular activation. We previously reported that the antibody-induced clustering of CLEC12A WT led to its co-localisation with flotillin-1 [17]. We confirmed this observation in Figure 4 that shows the presence of CLEC12A WT in the flotillin-enriched, insoluble fraction *(pellet)*. Similarly, cross-linking of the C130A mutant also induces the oligomerisation of a portion of the flotillin pool in the pellet that migrates at the same molecular weight as the CLEC12AWT and C130 oligomers Figure 4 *(lanes 5 and 7 in the right, Flot-1 and Flot-2 blots)*.

**Figure 8:**
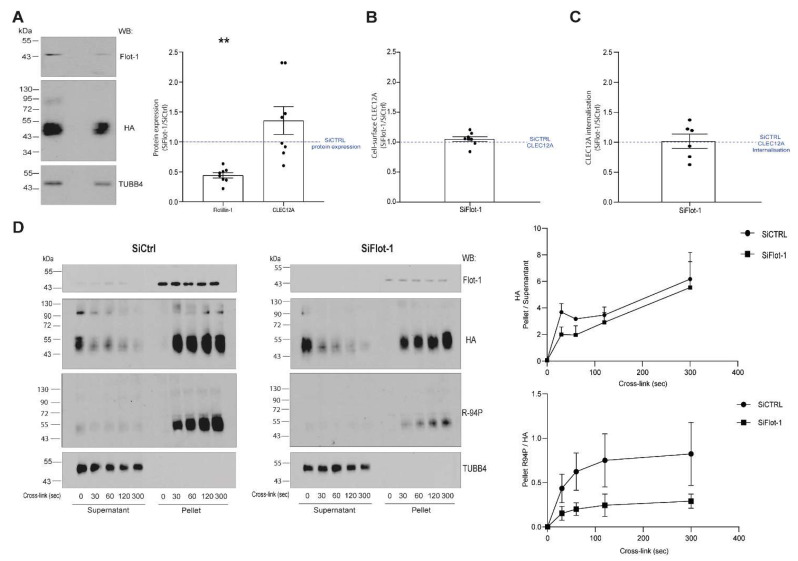
Flotillin depletion downregulates antibody-induced CLEC12A phosphorylation. (**A**) Flotillin-1 expression was knocked-down with a pool of flotillin-1-specific siRNAs *(siFlot-1)* in HEK-293T cells transiently transfected with CLEC12A WT. The diminution of flotillin-1 expression was confirmed by Western blot as was CLEC12A expression. (**B**) Cell-surface CLEC12A expression in flotillin knocked-down cells was compared to cells transfected with control (siCTRL) siRNAs by flow cytometry with an anti-HA antibody. The ratio of the MFI of the anti-HA signal in siFlot-1/siCTRL RNA-treated cells is shown in the graph. Data in (**A**,**B**) are representative of eight independent experiments. (**C**) The effect of SiFlot-1 on CLEC12A internalisation was determine by flow cytometry as described in ‘Materials and Methods’. Data are shown as the ratio of the median fluorescent index (MFI) of cell-surface CLEC12A in siFlot-1 cells/siCTRL cells after the antibody-induced internalisation of CLEC12A. These data are representative of eight independent experiments. (**D**) CLEC12A was cross-linked with the anti-HA antibody on HEK-293T cells transiently expressing WT CLEC12A and transfected with siFlot-1 or siCTRL siRNAs. CLEC12A translocation to the NP40-insoluble pellet and phosphorylation was analysed by Western blot of the supernatant *(SN)* and pellet *(P)* of cell lysates with the anti-HA and R-94P antibody. The anti-TUBB4 (tubulin 4) antibody was used as a loading control. These data are representative of four and three (for R-94P) independent experiments. Densitometric analysis was performed on the anti-HA data to calculate the ratio of CLEC12A in the pellet/supernatant in siFlot-1 and siCTRL siRNA-transfected cells *(top graph)*. The proportion of phosphorylated CLEC12A in the pellet is shown as the ratio of phosphorylated CLEC12A *(R-94P)*/non-phosphorylated CLEC12A *(anti-HA)* in siFlot-1 and siCTRL siRNA-transfected cells *(bottom graph)*. (**C**) Statistical analysis: One sample Wilcoxon test ** *p* < 0.01.

As our observations suggest that CLEC12A may oligomerize with flotillin after antibody-induced clustering, we explored the potential link between CLEC12A signaling and flotillin using a knock-down approach. The silencing of flotillin-1 expression in HEK-293T cells by 50% did not significantly alter CLEC12A cell-surface expression or its antibody-induced internalisation (Figure 8A–C). Likewise, the downregulation of flotillin-1 did not significantly decrease the antibody-induced translocation of CLEC12A WT to the NP40 insoluble pellet (Figure 8D, *anti-HA blot and top graph*). In contrast, silencing flotillin-1 expression significantly diminished the proportion of CLEC12A WT phosphorylated in the NP40 insoluble pellet (Figure 8D, *anti-R-94P blot and bottom graph*). Together, these observations suggest that, while flotillin-1 does not influence CLEC12A membrane expression or internalisation, it plays a key role in the ITIM phosphorylation required for receptor signaling following antibody-induced clustering of CLEC12A.

## 3. Discussion

Disulfide bonds are the most common covalent linkages in proteins [21]. They are particularly frequent in extracellular proteins such as CLRs whose CTLD fold depends on highly conserved cysteines [14]. In addition to the CTLD, some CLRs such as CLEC12A also habour cysteine residues in their stalk domain that are postulated to be essential for receptor oligomerisation [24]. The aim of this study was to elucidate the role of the stalk domain cysteines in CLEC12A expression and early signaling events. Our findings provide the first demonstration of the crucial roles for the two CLEC12A stalk cysteine residues in regulating receptor subcellular localisation, cell-surface expression and signaling. While the loss of C118 disrupts CLEC12A transport through cytoplasmic compartments to the cell-surface expression, mutation of C130 enhances CLEC12A oligomerisation and subsequently its phosphorylation and signaling. These findings are particularly pertinent to the modulation of myeloid cell activation by CLEC12A during inflammation due to the significant changes in the redox environment. In support of this notion, we show that CLEC12A oligomerisation is enhanced in the presence of hydrogen peroxide suggestive that the stalk cysteines have redox regulatory capacity. Moreover, we show that CLEC12A induces flotillin-1 oligomerisation and that flotillin is essential for CLEC12A signaling.

CLR stalk cysteine residues are mostly known for their role in receptor oligomerisation through the formation of disulfide bonds [24]. CLEC12A’s stalk cysteines are conserved with CLEC-1, a CLR that dimerizes through the formation of disulfide linkages in its stalk domain [25]. Similarly, the CLR LOX-1 that shares a high degree of homology with CLEC12A’s CTLD also forms homodimers through a disulfide bond formed by Cys140 within its stalk domain [26]. We provide direct evidence that C118 and C130 are also involved in disulfide bond formation as the dimerisation of the CLEC12A combinatorial mutant of both cysteines is significantly compromised. In contrast, the single C130A mutant enhances CLEC12A dimerisation and phosphorylation, and recruits SHP-2 to the flotillin-rich membrane, cellular fraction. We interpret this ligand-independent induced oligomerisation as a gain-of-function phenotype. A search of the the gnomAD database revealed a naturally occurring polymorphism that substitutes C130 with a tyrosine residue. Our data suggest that individuals with this polymorphism will have a constitutively active CLEC12A and a significant down-regulation of myeloid cell activation. Since CLR oligomerisation increases ligand binding affinity, the formation of CLEC12A oligomers due to the loss of C130 may further potentiate CLEC12A function through enhanced interactions with ligands. The C130Y polymorphism is more frequent in Asian than European populations suggestive that environmental and/or epigenetic factors may favor the retention of this polymorphism in a greater proportion of the Asian than the European population.

Cysteine residues ensure protein quality control through proper protein folding in the tightly controlled redox environment of the ER [19]. Naturally occurring mutations in membrane and secretory proteins causing ER retention and loss of protein function are common in genetic diseases including Pelizaeus–Merzbacher disease and von Willebrand’s disease [19]. While we provide evidence that loss of C118 disrupts CLEC12A’s transit through the secretory pathway significantly diminishing its cell-surface expression, naturally occurring polymorphisms at this residue have not been reported. It is highly likely that there is considerable selection pressure to avoid changes at this amino acid due to its crucial role in CLEC12A expression. To our knowledge, a role for stalk cysteine residues in CLR, cell-surface expression has not been previously reported.

In addition to naturally occurring polymorphisms, the redox status of the surrounding environment could modify CLEC12A function by affecting C118 and C130 formation of disulfide bonds due to changes in their thiol reactivity. Cysteines are highly reactive residues and they also play a role as redox molecular switches in addition to their oxidative protein folding properties in several proteins such as HMGB1 [19]. The function of this DNA-binding nuclear protein changes depending on the redox state of its cysteines. When fully reduced, HGMB1 promotes inflammation by activating cell migration and stimulating cytokine secretion. In contrast, sulphonylation inactivates HMGB1. Moroever, cysteines within the same protein may play differential roles in protein trafficking, dimerisation and function as reported for the HDL receptor, SR-B1 [27]. Our findings have implications for the role of CLEC12A in inflammation as we also show that hydrogen peroxide enhances CLEC12A oligomerisation. Whether C118 and C130 are equally reactive towards oxygen radicals remains unknown. The preferential oxidation of C118 would result in it forming a disulfide bond as shown in Figure 6 *(closed conformation)* and a gain-of-function phenotype. In contrast, oxidation of C130 would favor disulfide bonding at this residue as shown in Figure 6 and result in a diminution in receptor function. These two cysteines thus have distinct and counter-regulatory roles to ensure the appropriate post-translational processing and function of CLEC12A. While the presence of C118 ensures that CLEC12A’s transport from the ER to the plasma membrane is not hindered, the presence of C130 is necessary to regulate CLEC12A oligomerisation. Our data strongly suggest that CLEC12A stalk cysteines function as regulatory switches of CLEC12A cell-surface expression, oligomerisation and signaling. This regulatory function is highly likely influenced by oxidative environments.

We previously reported that CLEC12A co-localizes with flotillin in detergent-resistant membranes after antibody-induced cross-linking [17]. The current study shows that antibody-induced cross-linking of CLEC12A also induces flotillin-1 and flotillin-2 oligomerisation in the detergent-resistant membrane fraction. This is in line with previous reports that demonstrated the requirement of flotillin oligomerisation for its recruitment to these plasma membrane domains [28]. Consistent with the signaling promoting properties of flotillin-enriched membrane domains, a knock-down of flotillin-1 expression significantly diminished the phosphorylation of the CLEC12A ITIM. These observations underscore the crucial role of flotillin membrane domains in CLEC12A signaling. Flotillin interacts with a variety of receptors and signaling proteins explaining its involvement in a myriad of cellular processes including cell adhesion, endocytosis, phagocytosis and cell signaling [23,28]. Whether CLEC12A can also, in turn, regulate any of the diverse roles of flotillin through hetero-oligomerisation and/or altering flotillin phosphorylation remains to be determined.

While we provide evidence for a regulatory the role for Cys118 and Cys130 in CLEC12A expression, oligomerisaton and phosphorylation, the role of these residues in downstream CLEC12A signaling and function remains to be determined. Further experimentation will also reveal how flotillin and CLEC12A interact to regulate myeloid cell function.

In summary, our observations significantly further our understanding of CLEC12A function by identifying a crucial role for non-CTLD cysteines in regulating CLEC12A expression and signaling. Additionally, our data suggest that these cysteines act as redox-regulatory switches of CLEC12A signaling. Insight into how different inflammatory environments modulate CLEC12A expression and function through the stalk cysteines, will reveal how this myeloid inhibitory receptor contributes to the pathogenesis of autoimmune and inflammatory diseases.

## 4. Materials and Methods

### 4.1. Antibodies

Two different antibodies against the HA-tag were used, namely, the anti-HA.11 (mouse monoclonal 16B12; no. 90150) from BioLegend (Pacific Heights Blvd, San Diego, CA) and the rabbit polyclonal anti-HA (NB600-363B) from Novus biologicals (Oakville, ON, Canada). The former was used to cross-link CLEC12A-HA on HEK-293T cells and the latter, for immunoblotting. A mouse IgG1 isotype antibody (no. IM0571) was obtained from Beckman Coulter (Mississauga, ON, Canada) and used as a negative control.

The mouse IgG2a isotype control (no. 401502) antibody was obtained from BioLegend (Pacific Heights Blvd, San Diego, CA, USA). The affiniPure F(ab’)_2_ fragment goat anti-mouse IgG F(ab’)_2_ fragment specific (no. 115-006-072), the horseradish peroxidase-labeled donkey anti-rabbit IgG (no. 711-035-152), the horseradish peroxidase-labeled donkey anti-mouse IgG (no. 715-035-150) and fluorescein (FITC)-AffiniPure F(ab’)_2_ fragment goat anti-mouse IgG, Fcγ fragment specific (no. 115-096-071) antibodies were obtained from Jackson ImmunoResearch Laboratories (West Grove, PA, USA). The monoclonal anti-Flotillin-1 (no. 610820) and anti-Flotillin-2 (no. 610383) antibodies were purchased from BD Transduction Laboratories (Mississauga, ON, Canada) and phalloidin (no. A12381) from Thermofischer (Pacific Heights Blvd, San Diego, CA, USA). The mouse monoclonal anti-GAPDH (NBP1-47339) was obtained from Novus biologicals (Oakville, ON, Canada). Goat anti-mouse IgG (H + L) conjugated AlexaFluor 488 nm (A-11011) and the goat anti-rabbit IgG (H + L) conjugated AlexaFluor 594 nm (A-10012) antibodies were purchased from Invitrogen, Thermo Fisher Scientific (Waltham, MA, USA). The polyclonal anti-SHP-2 (sc-280) antibody was obtained from Santa Cruz (Dallas, TX, USA) and the mouse anti-human tubulin 4 (anti-TUBB4) (TUBB2C #WH0010383M2) antibody from Sigma-Aldrich Canada (Oakville, ON, Canada). The anti-phospho CLEC12A ITIM antibody (R-94P) was generated in-house and characterized in Paré et al. [20].

### 4.2. Reagents

Sodium orthovanadate (Na_3_VO_4_), trypsin inhibitor, PMSF, Nonidet P-40, Triton X-100, formalin 10%, aprotinin and leupeptin were obtained from Sigma-Aldrich Canada (Oakville, ON, Canada) and the Western Lightning Chemiluminescence Plus from PerkinElmer (Guelph, ON, Canada). Fetal bovine serum (FBS) and Dulbeco’s modified Eagle’s medium (DMEM) were purchased from Wisent Bioproducts (St-Bruno, QC, Canada). And Tween 20 as well as hydrogen peroxide (30%) from Fischer Scientific (Ottawa, ON, Canada). Polyethylenimine (PEI) was obtained from VWR (Mississauga, ON, Canada) and slowFade^TM^ Gold antifade reagent from Thermo Fisher Scientific (Waltham, MA, USA).

### 4.3. Plasmid Constructs and SiRNA Stealth^TM^ RNAi

The wild type, CLEC12A coding sequence used for our constructs corresponds to the CLEC12A isoform 2 sequence (Q5QGZ9-2 Uniprot)**.** The generation of the HA-tagged CLEC12A wild-type (WT) and CLEC12A-HA-Y7F construct with a mutated tyrosine in the ITIM motif (Y7F) were previously described in Paré [20]. To generate the CLEC12A-HA-C118A mutant construct with a mutated C118A, the open reading frame of CLEC12A-HA-wt was amplified with the forward; 5′ CAATAGCCACCAAATTAGCTCGTGAGCTATATAGC 3′ and reverse primer 5′ GCTATATAGCTCACGAGCTAATTTGGTGGCTATTG 3′. The PCR product was ligated to the pCRII plasmid using the same strategy as for CLEC12A-HA-wt. To generate the CLEC12A-HA-C130A construct with a mutated C130A, we used an overlap extension polymerase chain reaction (OE-PCR). The open reading frame of CLEC12A was amplified in two different PCR reactions with different pairs of primers. For the first PCR reaction for CLEC12A-HA-C130A we used the forward primer: 5′ AAGAGCACAAAGCTAAGCCTTGTC 3′ and reverse primer: 5′ TCTAGATGCATGCTCGAGCGGCCGCTTA 3′. For the second PCR reaction for CLEC12A-HA-C130A, we used the forward primer: 5′ CGCCAGTGTGCTGGAATTCTTTACATATT 3′ and reverse primer: 5′ GACAAGGCTTAGCTTTGTGCTCTT 3′. The two PCR products, one extending “upstream” and the other “downstream” of the desired mutation were mixed and hybridized while performing a third PCR using the oligonucleotides that targeted the 5′ and 3′ ends the hybrid PCR product, namely, the forward primer: 5′ CGCCAGTGTGCTGGAATTCTTTACATATT 3′ and the reverse primer: 5′ TCTAGATGCATGCTCGAGCGGCCGCTTA 3′. The PCR product was ligated to the pCRII plasmid using the same strategy as for CLEC12A-HA-wt. To generate the CLEC12A-HA-C118A/C130A double mutant, the open reading frame of CLEC12A-HA-C130A was amplified with the same primer and cloning strategy used to generate CLEC12A-HA-C118A. The SiRNA control (SiCTRL) (452001) and the SiRNA Flotillin-1 (FLOT1HSS115567) were obtained from Invitrogen.

### 4.4. Cell Culture and Transfection

HEK-293T and HeLa cells were maintained in Dulbeco’s modified Eagle’s medium (DMEM) containing 4 mM L-glutamine, 1 mM sodium pyruvate and 10% heat-inactivated fetal bovine serum. No antibiotics were used to culture these cell lines. Cells were seeded at a density of 0.3 × 10^6^ cells/well (HEK 293T or Hela) in 6-well plates the day prior to transient transfection of the CLEC12A-HA-WT or mutant plasmids (2 µg of DNA per well) with PEI as per the manufacturer’s instructions. The cells were harvested 48 h post-transfection with 10 mM EDTA in PBS prior to analysis.

### 4.5. Co-Transfection of Cell Lines with CLEC12A-HA-WT and the SiRNA Stealth^TM^ RNAi

Cells were seeded at a density of 0.2 × 10^6^ cells/well (HEK 293T) in 6-well plates the day prior to transient transfection of the CLEC12A-HA-WT plasmid (2 µg of DNA per well) and 20 mM SiRNA (SiCtrl or SiFlot-1) with PEI as per the manufacturer’s instructions. The cells were harvested 72 h post-transfection with 10 mM EDTA in PBS prior to analysis. To confirm flotillin-1 expression was downregulated, we performed a western blot for each experiment (western blot and flow cytometer) where flotillin-1 expression in SiFlot-1 was compared to SiCtrl to assess the SiRNA flotillin-1 downregulation.

### 4.6. Lysis of Transiently Transfected Hek 293T Cells Prior to Immunoblotting

HEK-293T transfected with CLEC12A mutant construct were harvested (10^6^ cells/100 µL PBS) and resuspended in the same volume of 2X modified Laemmli’s sample buffer reducing (composition of 1X: 62.5 mM Tris-HCl (pH 6.8), 4% (*w*/*v*) SDS, 8.5% (*v*/*v*) glycerol, 2.5 mM orthovanadate, 0.025% bromophenol blue, 10 μg/mL leupeptin, 10 μg/mL aprotinin, 5% (*v*/*v*) β-mercaptoethanol) or non-reducing (5% (*v*/*v*) β-mercaptoethanol replaced by water) and heated 95 °C for 7 min. When indicated cells were treated with pervanadate. Pervanadate was freshly prepared before use by mixing 1 mM orthovanadate, 0.03% hydrogen peroxide in H_2_O and then incubatingthe solution at room temperature for 15 min in the dark prior to the addition to cells 1 in 9, vol:vol dilution). Cells were incubated for 10 min at 37 °C in the dark prior to lysis in the same volume of 2X modified Laemmli’s sample buffer (see above) and immunoblotting.

When indicated, the CLEC12A-HA receptorswere cross-linked with an anti-HA, mouse monoclonal or the isotype antibody (3 µg/10^6^ cells) for 5 min at 37 °C and centrifuged prior to incubation with the goat anti-mouse F(ab’)_2_ anti F(ab’)_2_ antibody (3 µg/10^6^ cells) for 5 min at 37 °C. Cross-linking was stopped on ice and the cells centrifuged at 1000× *g* for 1 min. The cells were resuspended (10^6^ cells/100 µL PBS) and lysed with the same volume of 2X modified, reducing or non-reducing Laemmli’s sample buffer (see above). To prepare cell-free supernatants and pellets, cells were lysed in cold 1% NP40 and the cell pellet resuspended in cold 1% NP-40 lysis buffer (10 mM Tris-HCL pH 7.3, 137.2 mM NaCl, 1 mM EDTA, 10 µg/mL aprotinin, 10 µg/mL leupeptin, 2 mM sodium orthovanadate, 50 µg/mL trypsin inhibitor, 1 mM PMSF, 1% NP-40) and incubated for 10 min on ice prior to centrifuging at 15,000× *g* for 10 min at 4 °C. An aliquot of the supernatant (SN) was incubated at 95 °C for 7 min in the same volume of non-reducing or reducing, modified 2X Laemmli’s sample buffer. The pellets were washed with cold, 1% NP-40 lysis buffer and centrifuged at 15,000× *g* for 5 min at 4 °C. Pellets were sonicated with an ultrasonic pulse for 3 s prior to the addition of non-reducing or reducing, modified 2X Laemmli’s sample buffer and incubating at 95 °C for 7 min.

### 4.7. Electrophoresis and Immunoblotting

Proteins (20–25 µg/sample) were separated by SDS-PAGE on 10% acrylamide gels and transferred to PVDF membranes. Blocking agents and antibodies were diluted in a TBS-Tween solution (25 mm Tris-HCl, pH 7.8, 190 mm NaCl, 0.15% (*v*/*v*) Tween 20). Non-fat milk solution (5% *w*/*v*) was used to block nonspecific sites prior to immunoblotting with the anti-flotillin-1, anti-flotillin-2, anti-HA, anti-phospho CLEC12A (R-94P), anti-GAPDH and anti-SHP-2 antibodies. Anti-flotillin-1 [0.125 µg/mL], anti-flotillin-2 [0.125 µg/mL], anti-SHP-2 [0.2 µg/mL], anti-GAPDH [1 µg/mL], anti-TUBB4 (Tubullin 4) [0.5 µg/mL], rabbit anti-HA [1 µg/mL] and were diluted in TBS-Tween (0.15%). The anti-phospho CLEC12A ITIM antibody (R-94-P) was diluted at 0.4–0.8 µg/mL in TBS-Tween + BSA (5% *w*/*v*). Horseradish peroxidase-labeled donkey anti-rabbit IgG and horseradish peroxidase-labeled donkey anti-mouse IgG antibody were diluted at 50 ng/mL in TBS-Tween solution. Chemiluminescence reagents were used to detect antibodies within a maximal exposure time of 5 min. Equal protein loading was verified by immunoblotting against flotillin-1 or GAPDH.

### 4.8. Confocal Microscopy

Cells were transfected with the CLEC12A constructs 24 h after being seeded on coverslips (0.22 µm). Forty-eight hours post-transfection, cells were fixed with 9% formalin for 10 min at RT and washed with PBS prior to permeabilisation with 0.05% Triton X-100 in PBS for 10 min at room temperature. This was followed by an incubation for 20 min at room temperature in blocking solution (0.05% Triton X-100 in PBS supplemented with 4% FBS) and staining with the mouse anti-HA antibody [3 µg/mL for intracellular staining] and phalloidin AlexaFluor 594 nm in blocking buffer 30 min at 37 °C in dark. For extracellular staining, cells were incubated with a rabbit anti-HA antibody 7.5 µg/mL before fixation with formalin. Cells were then washed for 30 min at 37 °C and incubated with 5 µg/mL of secondary antibody against HA (anti-mouse AlexaFluor 488 nm) for 30 min at 37 °C in dark with blocking solution. After a wash in 0.05% PBS Triton X-100 for 30 min at 37 °C and water for 2 min followed by 1 min in 90% ethanol and 1 min in 99% ethanol, prior to mounting cells with SlowFade^TM^ Gold antifade reagent. No non-specific binding of the secondary antibodies was observed *(data not shown)*.

Images were acquired at 63X with a Z-stack spacing of 0.05 µm with the Quorum WAVFX spinning disc system (Quorum Technologies, Guelph, ON, Canada) and analyzed with the Volocity quantitation module. Briefly, confocal images were deconvoluted and the point spread function calculated for the GFP and Texas Red channel was applied using the velocity module for iterative restoration.

### 4.9. Quantitation of Antibody-Induced CLEC12A Internalisation by Flow Cytometry

HEK-293T cells transfected with the CLEC12A constructs were harvested (10^6^ cells/100 µL PBS) and incubated with anti-HA, mouse monoclonal or the isotype antibody (3 µg/10^6^ cells) for 5 min at 37 °C and centrifuged prior to cross-linking with the goat anti-mouse F(ab’)_2_ anti F(ab’)_2_ antibody (3 µg/10^6^ cells) for 5 min at 37 °C or incubated in buffer (no cross-linking). Cross-linking was stopped on ice and the cells centrifuged at 1000× *g* for 1 min. The cells were resuspended (10^6^ cells/100 µL PBS) prior to a 30 min incubation at 4 °C in the dark with an anti-mouse Fc-FITC conjugated (13 µg/mL) antibody. The cells were washed and resuspended in PBS and CLEC12A surface expression determined by flow cytometer.

### 4.10. H_2_O_2_ Treatment

HEK-293T transfected with CLEC12A WT were harvested with EDTA (10^6^ cells/100 µL PBS) and incubated in 100 µM H_2_O_2_ for 3 min or the same *v*:*v* with diluent (H_2_O). The reaction was stop by adding the same volume of 2X modified Laemmli’s sample buffer (see above) without beta-mercaptoethanol and boiled for 7 min at 100 °C.

### 4.11. Three Dimensional Modeling of CLEC12A

The 3D homology model (105–253 amino acid) of CLEC12A (Uniprot ID: Q5QGZ9) was constructed by the modeling software Modeller (Webb and Sali, 2014), using as templates from PDB database, 3g8l_A (105–125 amino acid) and 1yxk_B (126–253 amino acid). The homodimer was obtained by Galaxy homomer software (10.1093/nar/gkx246) (http://galaxy.seoklab.org/cgi-bin/submit.cgi?type=HOMOMER) (accessed on 1 February 2016). Coiled-coil of CLEC12A (68–88 amino acid) was determined by PCOILS (Gruber et al., 2006) and was added to dimer. Model quality was assessed by Ramachandran plot analysis through PROCHECK (Laskowski et al., 1993). Structure images were generated using PyMOL (http://www.pymol.org) (accessed on 1 February 2016).

## Data Availability

Not applicable.

## Supplementary Materials

The following are available online at www.mdpi.com/article/10.3390/ijms221910207/s1.
