# Supplementary material for: Regulation of the Expression, Oligomerisation and Signaling of the Inhibitory Receptor CLEC12A by Cysteine Residues in the Stalk Region
Source: Int J Mol Sci. 2021 Sep 22;22(19):10207. doi: 10.3390/ijms221910207 (PMC8508511; doi:10.3390/ijms221910207)
Supplement: Supplementary file 1 [file ijms-22-10207-s001.zip › ijms-1349923-supplementary.pdf]

Supplementary Materials:

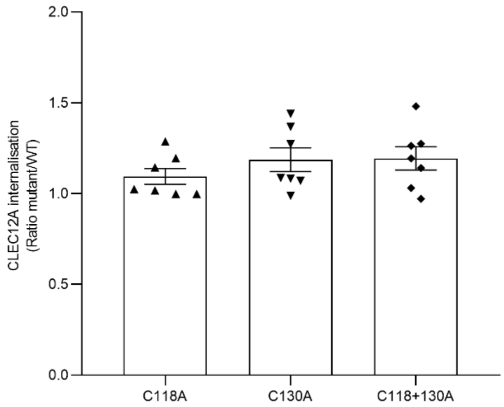

Supplemntary data Fig. S1: The effect of C118A and C130A on CLEC12A antibody-induced internalization.

HEK-293T cells transfected with CLEC12A constructs were cross-linked with a primary anti-HA. Cell-surface CLEC12A was then determined by staining cells with a secondary, anti-mouse Fc antibody prior to flow cytometry analysis as described in 'Materials and Methods'. Using the Median fluorescent index (MFI), the percent of each mutant internalization was calculated, and used to generated the mutant internalization ratio with CLEC12A WT basal internalization. These data are representative of 7 independent experiments. Statistical analysis: One sample Wilcoxon test * $P<0.05$.
